# Cancer genome and tumor microenvironment: Reciprocal crosstalk shapes lung cancer plasticity

**DOI:** 10.7554/eLife.79895

**Published:** 2022-09-08

**Authors:** Siavash Mansouri, Daniel Heylmann, Thorsten Stiewe, Michael Kracht, Rajkumar Savai

**Affiliations:** 1 Max Planck Institute for Heart and Lung Research Bad Nauheim Germany; 2 Institute for Lung Health (ILH), Justus Liebig University Giessen Germany; 3 Rudolf Buchheim Institute of Pharmacology, Justus Liebig University Giessen Germany; 4 Institute of Molecular Oncology Marburg Germany; 5 Member of the German Center for Lung Research (DZL) Giessen Germany; 6 Universities of Giessen and Marburg Lung Center (UGMLC) Giessen Germany; 7 Member of the Cardio-Pulmonary Institute (CPI) Frankfurt Germany; 8 Frankfurt Cancer Institute (FCI), Goethe University Frankfurt Frankfurt Germany; The Johns Hopkins University School of Medicine United States; Institute of Industrial Science, The University of Tokyo Japan

**Keywords:** cancer genome, tumor microenvironment, lung cancer

## Abstract

Lung cancer classification and treatment has been revolutionized by improving our understanding of driver mutations and the introduction of tumor microenvironment (TME)-associated immune checkpoint inhibitors. Despite the significant improvement of lung cancer patient survival in response to either oncogene-targeted therapy or anticancer immunotherapy, many patients show initial or acquired resistance to these new therapies. Recent advances in genome sequencing reveal that specific driver mutations favor the development of an immunosuppressive TME phenotype, which may result in unfavorable outcomes in lung cancer patients receiving immunotherapies. Clinical studies with follow-up after immunotherapy, assessing oncogenic driver mutations and the TME immune profile, not only reveal the underlying potential molecular mechanisms in the resistant lung cancer patients but also hold the key to better treatment choices and the future of personalized medicine. In this review, we discuss the crosstalk between cancer cell genomic features and the TME to reveal the impact of genetic alterations on the TME phenotype. We also provide insights into the regulatory role of cellular TME components in defining the genetic landscape of cancer cells during tumor development.

## Lung cancer

Lung cancer is the most frequently diagnosed cancer which also causes the highest burden of mortality amongst all malignant tumors worldwide (Siegel et al., 2021). Based on their histological features, lung tumors are divided into two major groups: (1) small cell lung carcinoma (SCLC), which accounts for 15% of all lung cancers, and (2) non-SCLC (NSCLC), which comprises 85% of all lung cancers (Travis, 2012). NSCLCs are further subclassified into adenocarcinoma, squamous cell carcinoma, and large cell carcinoma (Travis et al., 2015). The fact that a large group of patients has advanced disease stage at time of diagnosis explains the low 5-year survival rate of lung cancer patients, which decreases from 56% to only about 6% in the presence of metastasis (Siegel et al., 2019). This survival rate is the lowest compared with other common (metastatic) cancer types such as prostate, breast, and colorectal cancers (Bray et al., 2018). Originating from the global epidemic of tobacco consumption in the 20^th^ century, lung cancer was one of the main factors responsible for the increase of cancer-associated deaths observed within this period. However**,** during the last three decades, we have witnessed a significant decline in lung cancer mortality that can be attributed to (i) the reduction of smoking, (ii) the promising developments in early diagnosis and (iii) new therapeutic modalities (Siegel et al., 2021).

Broader public awareness of lung cancer and its relevant clinical signs as well as the initiation of novel screening concepts have proven that the frequency of devastating advanced stage disease can be reduced by earlier diagnosis (Jones and Baldwin, 2018). After diagnosis, improved video-assisted thoracoscopic surgery procedures to resect the tumors (Jones and Baldwin, 2018), as well as several new targeted medicines, including epidermal growth factor receptor (EGFR) tyrosine kinase inhibitors or immunotherapies are now available. In conjunction with classical cytotoxic chemotherapies (but also as stand-alone medications) these drugs have had a major impact on the reduction of lung cancer mortality rates in specific patient cohorts (Malhotra et al., 2017; Minguet et al., 2016).

## Genomic drivers of lung cancer cells

One of the main breakthroughs in cancer biology during the last three decades is related to the identification of disease-driving changes in proto-oncogenes and tumor suppressor genes. This has been accomplished by genome-wide profiling of the mutational landscape of cancer cells. It has also led to a reductionist view, in the sense that the central criterion to define a tumor are the genetic alterations found in cancer cells. Accordingly, a malignant tumor mass is (always) caused by genetically transformed cancer cells, which by their unlimited proliferation properties, fuel local and systemic cancer development and progression (Hanahan and Weinberg, 2000). Subsequently, a large body of evidence about key mutations has been obtained by whole genome DNA sequencing which has revolutionized our understanding of the genomic landscapes of cancer cells. These insights not only expanded lung tumor classification beyond histology, but also revealed that lung cancers belong to the most highly mutated tumors (Alexandrov et al., 2013). Specifically, profiling of the mutated genes of lung tumors revealed Kirsten rat sarcoma (*KRAS*), *EGFR*, anaplastic lymphoma kinase (*ALK*), *TP53* and liver kinase B1 (*LKB1*) as the most commonly mutated genes (Skoulidis and Heymach, 2019). In numerous clinical studies, the success of specific small-molecule inhibitors directed against some of the mutant oncoproteins finally demonstrated the importance of changes in the genetic landscape for the classification, pathogenesis and therapy of lung tumors (Skoulidis and Heymach, 2019).

## Lung cancer tumor microenvironment

However, the cancer-cell-based classification of any given tumor is too simplistic to explain or predict tumor behavior and its clinical response to treatment. A tumor is not just composed of genetically transformed cancer cells, but also contains multiple other types such as immune, stromal, and endothelial cells. This creates a unique environment, in which oxygen supply, availability of metabolites and pH are subject to tremendous fluctuations (Helmlinger et al., 1997; Brown and Wilson, 2004; Carmona-Fontaine et al., 2017; Altea-Manzano et al., 2020). Importantly, the non-transformed cells are not just passive bystanders, but have pivotal roles in tumor initiation, progression and metastasis (Hanahan and Weinberg, 2011; Baghban et al., 2020). Accordingly, the term tumor microenvironment (TME) has been introduced in the cancer field to indicate that non-transformed immune or stromal cells and their crosstalk with cancer cells not only regulate the tumor development at early stages of disease but also fulfill critical functions during advanced disease stages and metastasis (Binnewies et al., 2018).

## The modulation of the TME by the lung cancer cell genomic landscape

The TME is a double-edged sword that has anti-tumor activities early on but may promote tumor progression at later stages. In this regard, the cellular profile of the TME and its properties are key to defining the function of the TME as anti- or pro-tumor (Duan et al., 2020). The cellular TME components can be categorized into (i) pro-tumorigenic / immunosuppressive cells including pro-tumor M2-macrophages, myeloid-derived suppressor cells (MDSCs), and regulatory T (T_reg_) cells, and (ii) anti-tumorigenic immune effector cells, such as anti-tumor M1-macrophages, cytotoxic CD8^+^ T cells and natural killer (NK) cells (Duan et al., 2020; Zheng et al., 2017). The relevance of the interactions between these cell types and the tumor is evident from the remarkable anti-tumor effects of immune checkpoint inhibitors (ICIs). These therapeutic antibodies disrupt the negative regulation of T cell activity by cancer cells which is mediated by the interaction of programmed cell death ligand 1 (PD-L1, upregulated on cancer cells) with programmed cell death protein 1 (PD-1, (up)regulated on T cells) within the TME (Iwai et al., 2017). Although ICIs showed promising clinical outcomes in cancer patients, often only a subgroup of patients respond effectively (Robert, 2020). Therefore, there must be further mechanisms by which cancer cells not only suppress or hijack the TME antitumor functions but also impact the TME in a pro-tumor manner. Given the distinct genomic profile of cancer cells, it is plausible that the genetic landscape of cancer cells will impact on the TME phenotype and vice versa. Focusing on lung cancer, we will discuss new findings regarding the interplay of the genetic landscape of tumor cells and how it shapes the TME phenotype. We will also evaluate the crosstalk between the tumor genetic landscape and TME phenotype from a clinical point of view, addressing its potential application as a prognostic and/or therapeutic tool in lung cancer patients.

From the genetic point of view, the TME is a heterogeneous niche that contains a mixture of differentially mutated cancer cells, which give rise to the distinct cancer cell populations (clones) with their unique genomic landscapes (McGranahan and Swanton, 2017). Darwinian selection will lead to the preferential survival of clones with fitter phenotypes (Merlo et al., 2006). However, this type of selection can also be viewed as a ‘tragedy of the commons’ which shows how individuals driven by self-interest can be detrimental for the resource of the overall population. Based on this idea, the short-term interest of the selfish propagation in a distinct population (in this case a cancer cell clone with specific mutations) can be of ‘individual’ benefit. However, in the long term, it may also damage the cohabitants (in this case the TME with other clones and stroma / immune cells) and will finally lead to the destruction of the cohabitants and the extinction of the whole population (including cancer cells) (Hardin, 1968). Therefore, the fitness of selected clones cannot be solely defined from the perspective of cell autonomous growth as a consequence of the mutational landscape of cancer cells.

Interactions between organisms (i.e. cells) can be viewed as a game with multiple players and evolutionary game theory has been used to investigate the consequences of their interactions. According to the evolutionary game theory, the fitness of the selected cancer cell clones within the dynamic, complex and heterogeneous TME is not only associated with the individual cancer cell benefit but also essentially depends on the crosstalk of clones with other clones and cellular components of the TME (Archetti and Pienta, 2019).

**Figure 1.**
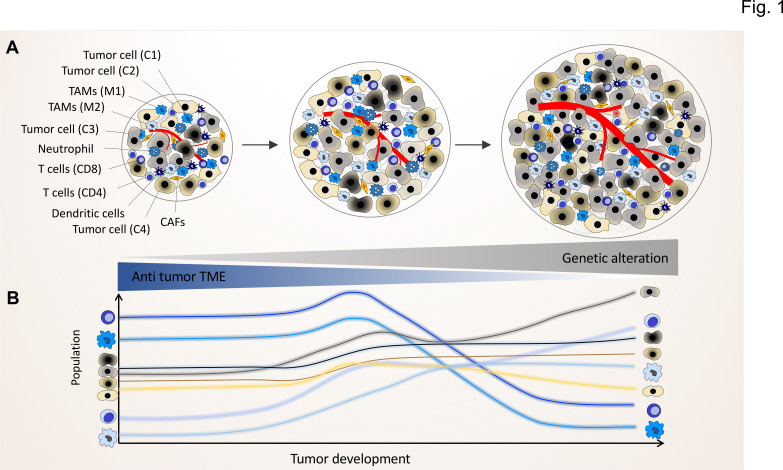
The genomic landscape of cancer cell defines the tumor microenvironment phenotype. Tumors contain various types of cancer cell clones during their development, which is depicted by different colored tumor cells (**C1, C2, C3, and C4**) to show the genetically distinct landscape. In the initial phase of tumor growth, the tumor microenvironment (TME) has a tumor-suppressive phenotype including mainly anti-tumor immune cells including M1-like TAMs and CD8^+^ T cells. During tumor progression, the TME phenotype reaches a steady state in which the population of anti-tumor and pro-tumor components (e.g. M2-like TAMs and CD4^+^ T cells) is in equilibrium. Based on evolutionary selection, the cancer cell clone with a unique genomic landscape and higher fitness (light gray clone) starts to re-shape the TME to enhance pro-tumor TME components that in turn support its growth and proliferation. In this process, the other less fitted clones are targeted by the anti-tumor TME elements and consequently depleted during tumor progression.

By applying the crosstalk scenario, we can advance the successive clonal evolution model of Peter Nowell, which did not fully consider the association of the non-genetic variability of immune / stroma cells and the potential functional crosstalk between clones and TME components (Figure 1; Janiszewska and Polyak, 2015; Nowell, 1976). A tumor arises in a multistep process, starting from a proliferating single-cell harboring genomic mutations and showing chromosomal and genetic instability within a unique normal microenvironment (NME), in which tissue homeostasis is tightly controlled through cellular crosstalk to maintain the balance of cell proliferation and death (Nelson and Bissell, 2006).

During the clonal evolution of the initial cancer cells, some of the daughter cells acquire stepwise additional somatic mutations. The sequential, subclonal selection not only favors cells with better autonomous proliferation capacities, but also selects cancer cells with the ability to transform the normal into a tumor microenvironment by reprogramming residential stroma or immune cells as well as newly recruited immune cells (Sun et al., 2018). Normally, immune or stromal cells of the NME impose protective constraints to prevent any disturbances in the balance of cell proliferation and death within the NME (Joyce and Pollard, 2009). Tumor growth will eventually depend on the success of cancer cells to alter the NME. If selected clones with specific TME-programming mutations can overcome the protective barriers of the NME, the tumor will eventually progress (O’Toole et al., 2014; Schietinger et al., 2016; Casanova-Acebes et al., 2021; Figure 1).

One argument supporting this concept is the observation that tumor doubling times (around 60–200 days) is orders of magnitude slower than cancer cell doubling times (around 1–2 days). This argues for a suppressive function of the NME and as a result the majority of cancer cells usually die before they can divide and establish a tumor mass (Klein, 2009; Greaves and Maley, 2012).

One of the key clinical observations in support of the relation between the genomic landscape of lung cancer cells and the TME is the association with tumor mutational burden (TMB), defined as the number of non-synonymous somatic mutations per megabase of tumor DNA, and the outcomes in patients treated with immunotherapy (Litchfield et al., 2021; Snyder et al., 2014). Recognition of cancer cells by immune cells mainly relies on presentation of tumor-specific antigens by the major histocompatibility complex (MHC) molecules on the surface of cancer cells which prime and activate the immune cells that can ultimately trigger cancer cell death (Schreiber et al., 2011).

Neo-antigens mostly originate from non-synonymous mutations. Despite the fact that not all somatic mutations will lead to neoantigens, TMB can serves as an indicator of neoantigen load of a tumor (Zou et al., 2021). In addition, common lung tumor mutations, including *KRAS* and *TP53,* are characterized by a high TMB and numerous somatic mutations, which are associated with increased tumor immunogenicity (Dong et al., 2017b; Gao et al., 2020). Thus, high TMB correlates with a greater probability of harboring neoantigens and cancer cell driver mutations (Chan et al., 2019). High TMB in turn will boost tumor immunogenicity, because more neoantigens are recognized by cytotoxic T cells, which promote the antitumor immune response (Rizvi et al., 2015). Given the central role of antigen/neoantigen for T cell activation, it is plausible that the TMB can guide immunotherapy-based strategies that rely on the activation of adaptive immunity (Sui et al., 2018).

Several retrospective studies indicated that tumor TMB is associated with the efficacy of immune checkpoint blockade and with clinical outcomes in lung cancer patients. In the KEYNOTE-158 study, tumor TMB-high status characterized a subgroup of patients with advanced solid tumors who showed a significant tumor remission in response to the anti-PD-1 monoclonal antibody pembrolizumab (Marabelle et al., 2020). In the CHECKMATE-026 study, the NSCLC patients were categorized in three groups based on TMB status (low TMB: 0 and 100 mutations, medium TMB: 100–242 mutations and high TMB: >243 mutations). High TMB patients treated with the anti PD-1 antibody nivolumab showed a longer progression-free survival (PFS; 9.7 vs 5.8 months; HR  = 0.62, 95% CI: 0.38–1.00) and higher response rates (47 vs 28%) than patients with the same TMB receiving chemotherapy. Moreover, patients with both high TMB and PD-L1 expression in more than 50% of the tumor cells had a higher response rate (75%) compared to patients with only one of these factors (32% among patients with a high tumor-mutation burden only and 34% among those with a PD-L1 expression level of ≥50% only; Carbone et al., 2016). In the CHECKMATE-227 study, patients with advanced NSCLC and a TMB status of at least 10 mutations per megabase, the combined treatment with nivolumab plus ipilimumab, an antibody targeting the global negative T cell regulator Cytotoxic T lymphocyte-Associated antigen (CTLA-4), resulted in a longer PFS compared to the chemotherapy group (7.2 vs 5.5 months; HR  = 0.58, 97.5% CI: 0.41–0.81, p < 0.001) (Hellmann et al., 2018).

Aneuploidy, the presence of an abnormal number of chromosomes in cancer cells (Holland and Cleveland, 2009), should also be considered, as a modulator of the TME immune-phenotype. Aneuploidy has been shown to be positively correlated with the number of mutations in the most types of cancer including lung tumor (Taylor et al., 2018). Three groups independently indicated that tumor aneuploidy negatively correlates with markers of immune evasion and number of tumor-infiltrating leukocytes (Davoli et al., 2017; Xian et al., 2021; Taylor et al., 2018). Davoli et al. reported a significantly reduced expression of genes associated with adaptive immunity, high cytotoxic activity mediated by CD8^+^ T cells and pathways related to the presence of pro-inflammatory cytokines in high aneuploid tumors. Moreover, they found highly aneuploid tumors to be associated with poorer survival of patients (Davoli et al., 2017). Xian et al., also showed that aneuploidy negatively correlated with immune-mediated cytotoxicity in most cancer types. The effect may be mediated by non-cell autonomous effects on immune cells including macrophages and T cells. Implicated in this process are polarized macrophages that support an immune suppressive phenotype and negatively regulate T cells during activation (Xian et al., 2021). Finally, Taylor et al., suggested that the negative correlation between aneuploidy and leukocyte infiltrates may explain the down-regulation of genes associated with immune signatures in high aneuploidy tumors.

These clinical studies provide evidence that the immune profile of the TME correlates with the genomic landscape of the lung tumor and suggest that future immune therapeutic strategies may be designed and applied in line with the TMB and aneuploidy. Although the correlation between tumor mutation load and immune therapy outcomes does not prove causality, clinical studies and new experimental evidence (see below) foster the idea that the cancer cell genomic landscape and the TME phenotype interact with each other.

## How the lung cancer cell mutational landscape shapes the TME phenotype

In the following, we will focus on specific lung cancer mutations in proto-oncogenes (KRAS, EGFR, ALK, MYC) and tumor suppressor genes (p53, LKB1) and how they relate to alterations in the TME. *KRAS* mutations are the most frequent oncogenic driver mutations in human lung cancer cells (Cancer Genome Atlas Research, 2014; Jordan et al., 2017; Skoulidis et al., 2015). Until very recently, no drugs were available to treat mutant KRAS-driven lung cancer (Huang et al., 2021). KRAS mutations not only cause cell autonomous proliferation and survival of cancer cells but also impact on the lung TME phenotype by non-cell autonomous modulation of immune cells (Dias Carvalho et al., 2018). Specifically, in lung cancer positive for oncogenic *KRAS* mutations, a pro-tumorigenic, immunosuppressed TME enriched in pro-tumor M2 macrophages, MDSCs, interleukin (IL)–17-producing T helper (Th)17 cells and CD4^+^FoxP3^+^ T_reg_ cells has been found (Cullis et al., 2018). This raises the question of the molecular links between KRAS and the altered TME.

KRAS is a small GTPase and an activator of MAPK pathways (Joneson et al., 1996). It has also long been known that mutant RAS proteins activate the nuclear factor of kappa light polypeptide gene enhancer of the B-cells (NF-κB) pathway in cancer cells (Cullis et al., 2018; Finco et al., 1997; Kim et al., 2002). The NF-κB family of transcription factors comprises five proteins whose homo- or heterodimers play important roles in regulating gene expression in many systems, including innate and adaptive immunity (Zhang et al., 2017). In most tissues, the nuclear activity of NF-κB subunits is tightly controlled by cytoplasmic retention through the binding to inhibitor of NF-κB (IκB) proteins (Oeckinghaus and Ghosh, 2009). In many tumors, the tight cytoplasmic control of NF-κB is relaxed, the pathway becomes constitutively (or chronically) activated and elevated levels of nuclear activity are observed (Taniguchi and Karin, 2018).

**Figure 2.**
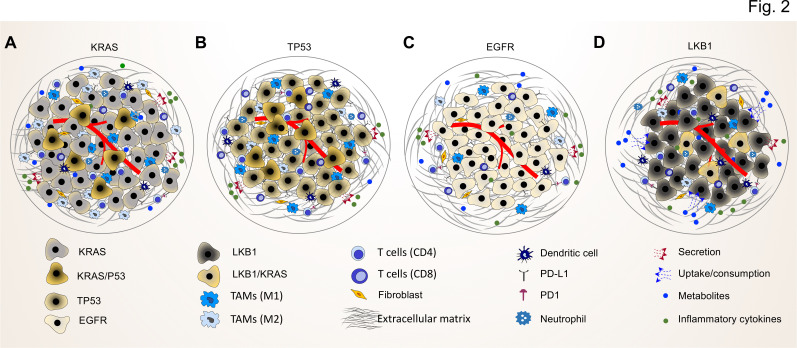
Common driver mutations impact on the TME in lung tumor. (**A**) *KRAS* mutations induce a pro-tumorigenic, immunosuppressed TME enriched in pro-tumor M2 macrophages and pro-tumor CD4^+^ T cells. *KRAS* activated intrinsic inflammatory signaling via the NF-κB pathway leads to increased secretion of inflammatory cytokines, including TNFα, IL-1α/β, and CXCL8, which in turn enforce the induction of a pro-inflammatory TME by polarization, and recruitment of immunosuppressive immune cells. (**B**) *TP53* mutations shape an inflamed and immunosuppressive TME via cell-autonomous activation of the NF-κB pathway in cancer cells and enhance the secretion of inflammatory cytokines such as interferon γ (IFNγ). Moreover, *TP53/KRAS* co mutated tumors express higher levels of PD-L1 and show higher CD8^+^ T cell infiltration. (**C**) *EGFR* mutated cancer cells have a lower mutational load. This unique genetic feature together with low level of PD-L1 expression and CD8^+^ tumor infiltrating T cells mark a unique immunosuppressive and cold TME phenotype, which can explain the resistance to immune-checkpoint therapies of lung cancer patients with *EGFR* mutation. (**D**) *LKB1* inactivation can lead to increased uptake of specific metabolites such as serine, which support immune cell activation and polarization. This can result in the extracellular depletion of immune regulatory metabolites and favor an immunosuppressive TME phenotype. *LKB1/KRAS* co-mutated tumors are further characterized by a pro-inflammatory cytokine milieu.

As NF-κB-binding sites are found in the enhancers and promoters of numerous immunoregulatory genes (Natoli et al., 2005), it is not surprising that NF-κB plays a pivotal role in cancer by fueling a pro-tumorigenic inflammatory environment through the induction of classical NF-κB target genes (Baud and Karin, 2009; DiDonato et al., 2012). These genes comprise a plethora of cytokines and chemokines, including IL6, tumor necrosis factor α (TNFα), IL-1α/β, CXCL1, 2, 5, and 8, monocyte chemoattractant protein 1 (MCP-1) / CCL2, and intracellular adhesion molecule 1 (ICAM1) as well as regulatory cell cycle and anti-apoptotic proteins (Baud and Karin, 2009; Eluard et al., 2020). The same factors are also upregulated in lung cancer and NF-κB is discussed for its role in driving the major hallmarks of NSCLC (Dimitrakopoulos et al., 2020; Figure 2). In the following, we discuss pivotal findings concerning KRAS-driven cytokine and chemokine networks in lung cancer.

By secreting IL-6, mutant KRAS cancer cells can activate Janus activated kinase 1 (JAK1) to induce the phosphorylation of the transcription factor signal transducer and activator of transcription 3 (STAT3) which is found in immune components such as macrophages of the TME (reviewed in Chonov et al., 2019). Studies in preclinical mouse models showed that the pharmacological blockade of IL-6 suppresses progression of KRAS-mutant positive lung cancer, inhibits STAT3 activation and more importantly diminishes the number of pro-tumor M2 macrophages, MDSCs and T_reg_ cells while the CD8^+^ T-cell responses increases (Caetano et al., 2016, Figure 2A).

CXCL8 /IL-8 is as chemokine that attracts polymorphonuclear inflammatory leukocytes to sites of tissue injury by acting on the chemokine receptors CXCR1/2 (Baggiolini, 1998; Harada et al., 1994; Russo et al., 2014). Tumors very frequently coopt the production of this chemokine to execute different pro-tumoral functions, including angiogenesis, survival signaling, and attraction of myeloid suppressor cells (Alfaro et al., 2017). Seminal work by Bar-Sagi et al. showed that mutant KRAS^G12V^ transcriptionally induces CXCL8 through the RAF-MAPK, PI3K and NF-κB signaling pathways (Sparmann and Bar-Sagi, 2004). Using a mouse xenograft tumor model, a neutralizing anti-CXCL8 antibody was shown to attenuate survival and neo-angiogenesis in a non-cell-autonomous manner and to reduce the infiltration of inflammatory immune cells (Sparmann and Bar-Sagi, 2004). This study demonstrated chemokine induced oncogenic activation of tumor cells that serves to regulate the TME. Likewise, the lung tissues derived from the Kras^G12D^ LA1 lung adenocarcinoma mouse model showed higher concentrations of the CXCL8 functional homologues KC and MIP-2 (Wislez et al., 2006). The reduced frequency and progression of lung tumors following antibody-based blockade of the CXCR2 receptor is further evidence for the role CXCL8 plays in tumorigenesis (Wislez et al., 2006).

IL-6, CXCL8 and multiple other chemokines (e.g. CXCL1, 2, 3, 5, CCL20, CCL2) are under complex control of ‘master cytokines’ such as IL-1α or IL-1β, which can induce the transcriptional and post-transcriptional expression of all of these factors simultaneously through a network of signaling pathways and transcription factors (including the NF-κB system) in multiple normal as well as cancer cells from both mice or humans (Gaestel et al., 2009; Jurida et al., 2015; Weber et al., 2010; Wolter et al., 2008; Ziesché et al., 2013). The IL-1 system can be successfully pharmacologically targeted in a range of chronic inflammatory conditions by the (i) IL-1 receptor antagonist (IL-1RA), (ii) the fully human anti IL-1β antibody canakinumab, or (iii) a recombinant IL-1 receptor IgG fusion protein called rilonacept (Dinarello et al., 2012; Mantovani et al., 2019a). Of note, the IL-1RA is equally effective in suppressing IL-1α and IL-1β, while canakinumab is IL-1β-specific (Dinarello et al., 2012).

IL-1α/β have also long been considered as crucial cytokines in cancer, however often contradictory pro- or anti-tumorigenic roles were found in different tumor models or clinical studies (Garlanda and Mantovani, 2021). While both cytokines activate the same IL-1 receptor heterodimer, composed of IL-1R1 and IL-1R3 (also called IL-1R accessory protein, IL-1RAcP), and are supposed to initiate the same downstream gene-regulatory effects (see above), they differ in their post-transcriptional processing and release mechanisms. The IL-1α precursor can function intracellularly as an active nuclear transcriptional regulator and the processed mature IL-1α is primarily membrane-associated or released locally from severely damaged, necrotic cells (Kim et al., 2013; Malik and Kanneganti, 2018; Rider et al., 2013; Werman et al., 2004). In contrast, the inactive IL-1β precursor requires processing by the inflammasome complexes as well as secretion to induce local but also systemic inflammation (Mangan et al., 2018). The Apte lab systematically studied the role of IL-1α or IL-1β in tumor initiation and progression using several genetically engineered mouse models. They showed that nuclear IL-1α acts as a signal for genotoxic stress and binds to sites of DNA damage and thereby regulates tissue inflammation (Cohen et al., 2015). They also assessed the role of IL-1β and IL-17, an IL-1-inducible T cell cytokine, in a model of experimental lung metastasis. Using IL-1β or IL-1RA-deficient mice, they found that lack of IL-1β or excess of IL-1 activity (by lack of IL-1RA) in the lung TME both resulted in reduced T cell activity and a poor prognosis. IL-1β-deficient mice showed increased T_reg_ numbers and activities whereas in IL-1RA-deficient mice enhanced accumulation and activity of myeloid-derived suppressor cells were found, both resulting in suppressed antitumor immunity. In mice lacking IL-17, the study showed reduced tumor progression along with improved T cell function. These data provided evidence for a critical and unique role of IL-1 in upregulating cytokines (e.g. IL-17) and in determining the balance between inflammation and antitumor immunity in specific tumor microenvironments such as in the lung (Carmi et al., 2011).

Further studies corroborate the importance of the IL-1/IL-17 axis also for KRAS-driven lung cancer. KRAS mutations in lung epithelial cells lead to the recruitment of Th17 cells and increased IL-17 production, both of which are associated with lung tumorigenesis (Chang et al., 2014). IL-17 secreted from tumor-infiltrating Th17 lymphocytes can induce the epithelial-mesenchymal transition (EMT) gene-regulatory events in lung cancer cells, thereby promoting tumor cell migration, intravasation, and metastasis (Salazar et al., 2020). Accordingly and in line with the results from Apte et al., the blockade of IL-17 in mouse cancer models results in a reduction of lung tumor metastasis and the genetic ablation of IL-17C in a *Kras*^G12D^ lung cancer mouse model improved the response to anti-PD1 treatment (Ritzmann et al., 2019), (Chang et al., 2014; Salazar et al., 2020; Figure 2A). Moreover, the numbers of Th17 lymphocytes in patients with lung cancer negatively correlate with overall survival (Salazar et al., 2020).

Based on their own results and observations from other studies, Apte et al. concluded that IL-1α and IL-1β play opposing roles in the malignant process. While the membrane-associated IL-1α is mainly immunostimulatory, IL-1β that is secreted into the TME is mainly pro-inflammatory and promotes tumorigenesis, tumor invasiveness, and immunosuppression. These distinct functions of the two IL-1 agonists are important in early stages of tumor development and contribute to tumor progression according to their expression patterns within the TME (Voronov and Apte, 2017). New data provide further support for this concept. In a murine breast cancer model, IL-1β-deficiency resulted in IL-12 secretion by CD11b^+^ dendritic cells (DCs) cells and supported antitumor immunity by activated CD8^+^ lymphocytes expressing IFNγ, TNFα, and granzyme B. These T cells infiltrated tumors and induced their regression. Treating mice first with anti-IL-1β antibodies followed by anti-PD-1 antibodies completely abrogated tumor progression. These data define microenvironmental IL-1β as a master cytokine in tumor progression whose suppression also facilitates successful therapeutic checkpoint inhibition (Kaplanov et al., 2019).

These pre-clinical observations gained new importance with results emerging from the Canakinumab Anti-inflammatory Thrombosis Outcomes Study (CANTOS) trial (Ridker et al., 2017a). The CANTOS trial recruited 10,061 patients who had a previous myocardial infarction and some type of low level, smoldering inflammation (baseline CRP levels of ≥2 mg/L). Patients were treated with optimal medical and lipid-lowering therapy, and randomized to receive canakinumab or placebo (Ridker et al., 2017a). The CANTOS trial was designed to proof the concept that targeting IL-1β-mediated chronic inflammation can reduce cardiovascular events. Unexpectedly, the prespecified safety analysis of the trial data also revealed that treatment with canakinumab was associated with a dose-dependent reduction in lung cancer incidence (HR = 0.33; 95% CI, 0.18–0.59; p<0.0001 for the canakinumab 300 mg group compared with placebo) as well as lung cancer mortality (HR = 0.23; 95% CI, 0.10–0.54; p<0.0002 for canakinumab 300 mg group compared with placebo) (Crossman and Rothman, 2018; Ridker et al., 2017b).

A recent follow-up study examined circulating tumor DNA (ctDNA) and nine soluble inflammatory biomarkers (CRP, IL-6, IL1RA, IL-18, Leptin, TNFα, adiponectin, fibrinogen, and PAI1) in blood samples from CANTOS patients. Catalogue of Somatic Mutations in Cancer (COSMIC) database ctDNA mutations were detected in 65% (46/71) of the CANTOS patients with lung cancer but none of the mutations commonly found in lung cancer were enriched (or depleted) following canakinumab treatment. Further, median time to lung cancer diagnosis was shorter in patients with (n=29, 407 days) versus without (n=38, 837 days) detectable COSMIC ctDNA mutations at baseline (p=0.011). High baseline levels of CRP and IL-6 trended toward shorter median time to lung cancer diagnosis, suggesting that IL-1β-inducible CRP and IL-6, similar to ctDNA at baseline, correlate positively with a more rapid progression to lung cancer diagnosis (Wong et al., 2020). These results provide further evidence for the importance of the IL-1β pathway for pro-tumor inflammation in lung cancer and suggest canakinumab’s effect may be mediated by delaying inflammation-driven disease progression of diverse molecular subtypes of lung cancer.

In conclusion, the results from CANTOS as well as the findings from pre-clinical studies clearly point to an important role of the IL-1 system, autocrine or paracrine cytokine loops, and the downstream cytokine / chemokine networks for lung tumor development and progression (Briukhovetska et al., 2021; Yang et al., 2021).

*EGFR* is also one of the most commonly mutated genes in lung cancer. EGFR is a trans-membrane glycoprotein with an extracellular ligand binding domain for epidermal growth factor, a transmembrane domain, and an intracellular tyrosine kinase domain that regulates epithelial tissue maintenance and growth (Liu et al., 2017). The clinical outcomes of immune checkpoint therapies have revealed that *EGFR* mutations in lung cancer cells can not only upregulate the intrinsic PD-L1 expression on cancer cells but also suppress T cell function and increase levels of pro-inflammatory cytokines within the TME, which marks an immune escape phenotype of *EGFR*-mutant NSCLC (Akbay et al., 2013; Chen et al., 2015). Moreover, it has been shown that patients with *EGFR* mutations have fewer T cell infiltrations of PD-L1^+^/CD8^+^ tumor infiltrating lymphocytes (TILs) and reduced shrinking properties of the tumor in response to immune cell activation (Figure 2C, Dong et al., 2017a). In comparison to the reduced number of cytotoxic T cells in EGFR-mutant lung adenocarcinomas, patients with oncogenic EML4-ALK rearrangements showed a significant increase in regulatory T cells, supporting the concept that different driver oncogenes induce distinct immunosuppressive mechanisms (Budczies et al., 2022).

The *MYC* oncoproteins belong to a super-transcription factor family that regulates the transcription of at least 15% of the entire genome (Dang et al., 2006). *MYC* activation is involved in the regulation of cell-cycle progression, apoptosis, cellular senescence, and metabolism (Gabay et al., 2014). Co-occurrence of *MYC* genetic alterations with *KRAS* mutations considerably accelerates lung tumor development with a reduction of the survival rate in mouse lung tumor models (Kortlever et al., 2017). This mutational combination not only generates highly proliferative, invasive cancer cell clones but also reprograms the TME towards the inflammatory, angiogenic, and immune-suppressed phenotype. Mechanistically, *MYC*-induced immunosuppression relies on CCL9 and IL-23. CCL9 regulates the recruitment of CD206^+^ pro-tumor macrophages and PD-L1-dependent expulsion of T and B cells. IL-23 boosts the pro-tumor CCL9 effects through the elimination of adaptive T and B cells and innate immune NK cells (Kortlever et al., 2017). Blocking of CCL9 and IL-23 abrogates the lung tumor aggressiveness in *KRAS/MYC-*altered mice mainly by re-establishing a tumor-suppressive lung microenvironment (Kortlever et al., 2017).

*TP53*, encoding the tumor suppressive transcription factor p53, is not only the most frequently mutated genes in cancer but also across all types of lung cancer with mutations rates ranging from 46% in lung adenocarcinoma to over 90% in SCLC (Gibbons et al., 2014; George et al., 2015). *TP53*-mutant tumors show a distinct TME profile including increased PD-L1 expression and CD8^+^ T cell infiltration, which suggest an adaptive immune resistance and a high immunogenicity state (Dong et al., 2017b). The excess of *TP53* mutations in lung cancer cells can also elevate NF-κB activity (Scian et al., 2005). Interestingly, enhanced activation of NF-κB suppresses p53-mediated gene activation and thereby promotes resistance to apoptosis in cancer cells (Webster and Perkins, 1999). Similar to *KRAS* mutations, *TP53* mutation-mediated NF-κB activation has an additional, non-cell autonomous impact on the TME through the secretion of a variety of pro-tumor and immunosuppressive cytokines and chemokines (Figure 2B; Mantovani et al., 2019b). In *KRAS*-mutant lung cancer cells with *TP53* co-mutations, increased NF-κB signaling conferred pro-survival signals to the cancer cells (Meylan et al., 2009). With regard to TME phenotype, *KRAS/TP53* lung tumors are further characterized by increased expression of PD-L1 and a higher proportion of PD-L1^+^/CD8a*^+^* T cells compared to *KRAS* or *TP53* single mutation (Dong et al., 2017b). Moreover, *TP53* mutations also directly impact on the protein secretion machinery itself (Pavlakis and Stiewe, 2020; Pavlakis et al., 2020). For example, *TP53* inactivation in lung adenocarcinomas was found to activate progestin and adipoQ receptor 11 (PAQR11)-mediated prometastatic secretory vesicle biogenesis in the Golgi, resulting in the autocrine activation of a PLAU receptor/STAT3/PAQR11 feedforward signaling loop that triggers an immunosuppressive TME rich in effector/memory CD8^+^ T cells and M1 macrophages (Tan et al., 2021a). Similarly, *TP53* loss induces Golgi reassembly and stacking protein 55 kD (G55)-dependent secretion that promotes angiogenesis and CD8^+^ T cell exhaustion (Tan et al., 2021b). Missense mutations in *TP53* specifically induce the endoplasmic reticulum UDPase ENTPD5 that drives the calnexin/calreticulin cycle required for proper folding of secreted proteins (Vogiatzi et al., 2016). The loss of *TP53* or the expression of mutant p53 proteins thereby acts at multiple non-cell-autonomous levels to blunt antitumor and promote tumor-supporting TME properties (Blagih et al., 2020).

Co-mutation of *KRAS* and the tumor suppressor *LKB1*, one of the most prevalent mutational combinations in lung tumor, is observed in about ~25% of *KRAS*-mutant lung adenocarcinomas (Skoulidis et al., 2015). *LKB1* is a serine-threonine kinase, which has important regulatory roles in cellular metabolism and energy stress response mainly through activating AMP kinase (AMPK) and AMPK-related family members (Marcus and Zhou, 2010). Hence, mutations in *LKB1* have tremendous impact on energy and metabolic profiles of a stressed TME, wherein cancer cells and immune or stromal cells are subjected to metabolic alterations, limited nutrient availabilities, hypoxia, and pH disturbances (Zheng et al., 2020a). Mutational cooperation between *LKB1* loss and *KRAS* activation leads to induction of the serine–glycine–one-carbon pathway in lung cancer cells which results in enhanced *S*-adenosyl methionine (SAM) synthesis as a critical substrate for DNA methylation. Upregulation of SAM leads to increased DNA methylation in lung cancer cells with *KRAS/LKB1* co-mutations (Kottakis et al., 2016). Interestingly, extracellular serine within TME is essential for optimal T cell expansion through supplementation of glycine and one-carbon units for de novo nucleotide biosynthesis in proliferating T cells. Accordingly, upregulation of the serine–glycine–one-carbon pathway in *LKB1* mutated cancer cells can deplete the serine level in the extracellular space and thereby regulate T cell proliferation and function (Ma et al., 2017). Furthermore, *KRAS/LKB1* co-mutated lung tumors are characterized by an altered nitrogen metabolism. *KRAS/LKB1*-mutant lung cancer cells are more dependent on an unorthodox pathway of pyrimidine biosynthesis that utilizes mitochondrially generated carbamoyl phosphate through upregulation of carbamoyl phosphate synthetase-1 (CPS1; Kim et al., 2017). CPS1 allows the mutated cancer cells to become resistant to arginine depletion, which is a major strategy of pro-tumor macrophages and tumor-associated myeloid cells to inhibit antigen-specific T cell responses (Bronte and Zanovello, 2005). Along the same line, primary resistance to PD-1 based therapies in *KRAS*-mutant lung cancer patients is mainly associated with *LKB1* alterations in cancer cells. Furthermore, the genetic ablation of *Lkb1* also induces the resistance to anti–PD-1/anti–PD-L1 therapies in murine *Kras*-mutant lung cancer models (Skoulidis et al., 2018). Therefore**,** *KRAS/LKB1* mutations in cancer cells not only establish a non-T cell, inflamed TME including reduced infiltration of CD3^+^, CD4^+^, CD8^+^ T cells and low expression of PD-1, but also help the cancer cells to cope with harsh conditions in the TME, when metabolites such as arginine become limited depending on the immune status (Figure 2D).

## How the tumor microenvironment shapes the genomic landscape of lung cancer cells

**Figure 3.**
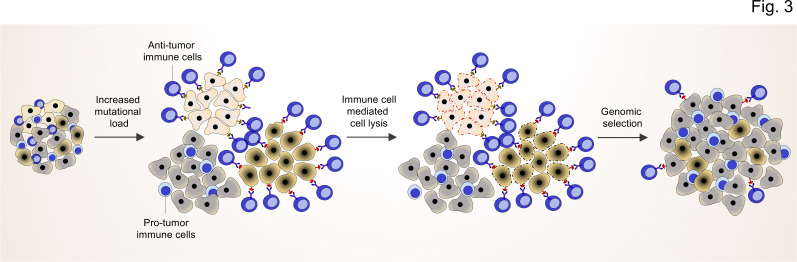
TME shapes the genomic landscape of lung cancer cells. Accumulation of specific cancer clones (light and dark cream) with (neo)antigen presentation can induce the infiltration of anti-tumor immune cells (blue cells with light nuclei). The anti-tumor immune cells will recognize and induce the cell death pathway in the targeted clones. By this mechanism, anti-tumor immune cells can act as a genetic selection barrier not only by killing less fit clones but also through the preparation of an environment that promotes growth and proliferation of other clones (dark gray) with specific genetic profiles that subsequently prevent antigen production. However, some cancer cells with antigen presentation can also evade the killing by anti-tumor immune cells through other mechanisms (dark cream clones after genomic selection).

The mutational characteristics of cancer cells can shape the cellular composition of the TME, in particular the immune phenotype, but there is less attention paid to the fact that the immune components of the TME can vice versa modify the tumor genomic landscape. As tumorigenesis is an evolutionary process of clonal selection, cancer cell clones with strongly immunogenic neo-antigens are susceptible to recognition and elimination by immune cells at the early stages of tumor development. This can confer an evolutionary advantage to tumor development through the elimination of those clones with high antigenic mutations or aberrations (Figure 3). Simultaneously, other clones that manage to restrict neo-antigenic peptide presentation by MHC I molecules become invisible to the adaptive immune system and evade the anti-tumor immune response. Accordingly, the immune phenotype of the TME can shape the genomic landscape of tumor not only by detecting and eliminating immunogenic clones but also by promoting the outgrowth of clones that can evade immune responses. The important role of immune cells is underlined by the increased tumor susceptibility of immunodeficient mice compared to wild-type mice and the higher immunogenicity of cancer cells from immunodeficient mice (Koebel et al., 2007). Also, it has been documented that immunosuppressed transplant recipients with kidney failure developed secondary tumors after transplantation from undetected (occult) cancer of the organ donors (MacKie et al., 2003).

More recently, Marty et al. developed the Patient harmonic-mean best rank (PHBR) score to predict the probability for a patient’s MHC I variants to bind to a peptide sequence containing the relevant residues which can be recognized by matching immune cells. In their model, low PHBR scores demonstrate a high likelihood of residue presentation (Marty et al., 2017). Interestingly, mutations in known oncogenes and tumor suppressor genes have higher PHBR scores than random mutations, which means less recognition probability by immune cells. Moreover, they could find a positive correlation between the PHBR score of any given mutation and the frequency of the mutation in tumors. Accordingly, an oncogenic mutation can acquire the high mutation frequency not only because of a cell-autonomous fitness benefit provided by the mutation but also due to poor presentation of the mutant peptide, which renders the mutations undetectable to the immune cells (Marty et al., 2017).

Furthermore, McGranahan et al. revealed that tumors with clonal loss of heterozygosity (LOH) in the HLA locus, the gene which encodes MHC I molecules, is associated with a high neoantigen burden, APOBEC-mediated mutagenesis and significantly elevated PD-L1-positive immune cells compared to tumors without any HLA LOH, suggesting that HLA LOH can be considered as an immune escape mechanism (McGranahan et al., 2017). The authors proposed that infiltration of immune cells, including CD8^+^ T cells, happens upon the accumulation of antigen/neoantigen within cancer cells. This immune infiltration creates a selection barrier for tumors by eliminating the clones with high antigen/neoantigen load. However, cancer cell subclones with HLA LOH may be positively selected based on their evasion capability from CD8^+^ T cell recognition (McGranahan et al., 2017).

Altogether, the development of the mutational landscape of cancer cells in the early stage of tumor development is tightly interconnected with the immune components of the TME. Although the immune component of TME can recognize and eliminate the clones with high antigen/neoantigen load, they eventually co-determine the genomic landscape of tumor by paving the way for the fitter clones with less immunogenic mutations (lower PHBR scores) or antigen presentation defects (high HLA LOH), which supports evasion from the immune response (Figure 3).

## New and advanced approaches to study the TME

As outlined above, there is ample evidence to suggest that cytokine networks govern immune evasion and foster the development of immunosuppressive T cells and TAMs within the TME (Van Den Eeckhout et al., 2021; Van Den Eeckhout et al., 2020). However, it is still unclear how to tackle the TME cytokine milieu therapeutically in order to trigger strong and sustained antitumor responses in the majority of patients. A large and comprehensive survey of completed and ongoing clinical trials on cytokines and chemokines concluded that, with few exceptions in small numbers of patients, inhibiting or enhancing a single cytokine or chemokine pathway is unlikely to have sustained activity against advanced-stage cancer (Propper and Balkwill, 2022). Thus, while new pre-clinical and clinical results reinforce the idea that cytokines and chemokines should be targeted to reprogram the TME as outlined above, new strategies are needed for effective therapies.

With respect to the TME, it is important to consider the local concentrations of cytokines at time of diagnosis (and biopsy) and how they may change over time (during therapy). Many cytokines have natural antagonists and the ratios of agonists to antagonists will determine the TME phenotype. For example, the IL-1 family of cytokines (IL-1F) comprises seven proinflammatory receptor agonists (IL-1α/b, IL-18, IL-33, IL-36 α/β/γ) and four anti-inflammatory or antagonistic members (IL-1RA, IL-36Ra, IL-37, IL-38) (Teufel et al., 2022). Most cytokines regulate gene expression patterns in their target cells (Gaestel et al., 2009). It is therefore important to consider the cytokine-activated effects downstream of their receptors and the cell-to-cell heterogeneity of these effects within the TME in order to understand the outcome of systemic manipulations of individual cytokines or their neutralization in patients.

**Figure 4.**
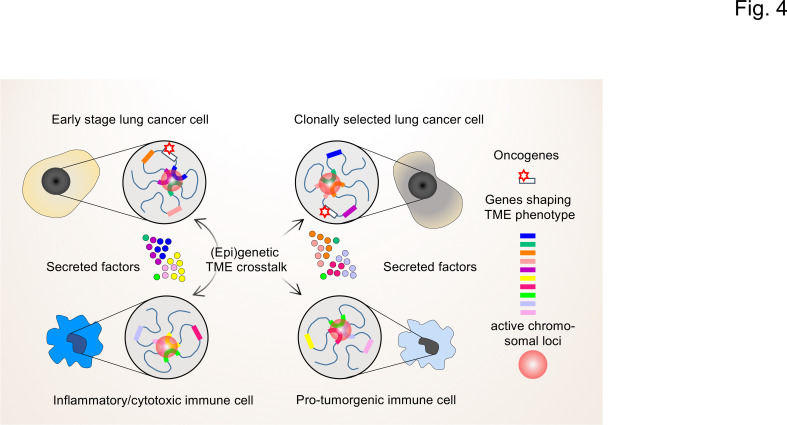
The interplay of single-cell chromatin states determine changes in gene expression in the TME of lung cancer cells. At the single cell level, gene expression occurs in a stochastic fashion and stable phenotypes in populations of tumor cells or immune cells result from variable single-cell gene expression patterns. Depicted are changing patterns of active chromatin loci formed by transient interactions of enhancers and promoters of multiple genes, some of which may encode secreted factors secreted by the TME. These nuclear foci operate as transcriptional hubs that are characterized by high concentrations of transcription factors, transcriptional cofactors and RNA polymerases. Similarly, the 3D spatial conformations of chromatin (including the loops that engage in enhancer promoter interactions) vary from cell to cell. This high degree in (epi)genetic diversity very likely contributes significantly to the clonal selection of cells that shape the TME in a pro-tumorigenic manner.

Towards this goal, we propose that it will be instrumental to determine the (epi)genetic profile of individual cells in the TME by combinatorial high-resolution approaches to map cell states and understand the regulatory diversity at the single cell and molecular levels (Shema et al., 2019). While the overall gene expression pattern of a population of (tumor) cells might appear stable, at the single-cell level gene expression occurs stochastically with individual genes undergoing cycles of bursts in activity and periods of inactivity (Misteli, 2020). This type of gene activity is closely linked to the different levels of 3D hierarchical genome organization within the nucleus (chromosomes, compartments, topologically-associating domains (TADs), DNA loops, nucleosome accessibility; reviewed in Finn and Misteli, 2019). High-throughput chromosome conformation capture (Hi-C) and high-throughput imaging assays can now be used to systematically map chromatin states of individual cells (Finn et al., 2019). Together with the multitude of next generation sequencing (NGS) techniques now available to measure mRNA expression and chromatin accessibility at single cell resolution, the genetic activities of individual cells can be precisely mapped (Kaya-Okur et al., 2019; Luecken and Theis, 2019; Meers et al., 2019; Wu et al., 2021). The basic concepts of these approaches are summarized in Figure 4.

The application of multi-level single-cell NGS approaches will allow to track the genetically most active cells within the TME and to monitor their functional changes upon tumor progression or during therapy (Guruprasad et al., 2021; Longo et al., 2021; Shema et al., 2019). For example, in a recent study whole-exome and transcriptomic data for >1000 immune checkpoint inhibitor-treated patients across seven tumor types were combined with single-cell RNA-seq data from clonal neoantigen-reactive CD8^+^ tumor-infiltrating lymphocytes (TILs), to identify CCR5 and CXCL13 as T cell-intrinsic markers of ICI sensitivity (Litchfield et al., 2021).

**Figure 5.**
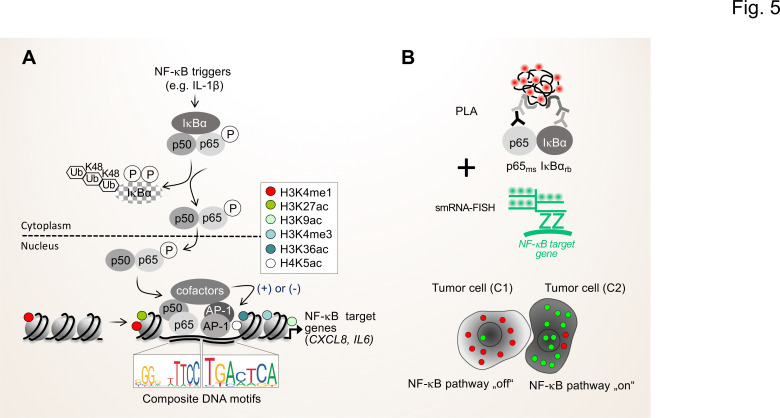
Single cell states of the NF-κB signaling pathway. (**A**) The scheme shows the key steps of the NF-κB signaling system. Activation of the canonical NF-κB pathway by triggers such as IL-1β will lead to the rapid phosphorylation and ubiquitination-mediated destruction of the cytosolic inhibitor IκBα. This in turns liberates the active NF-κB transcription factor subunits p50 (encoded by the *NFKB1* gene) and p65 (encoded by the *RELA* gene), which translocate to the nucleus. The p50 and p65 dimers bind to specific DNA motifs within accessible, open chromatin regions, often in conjunction with other transcription factors such as JUN or FOS proteins which form activating protein (AP)–1 (hetero)dimers. Many NF-κB target genes encode secreted factors such as *IL-6* or *CXCL8*, which are crucial regulators of the TME. High activity of NF-κB target genes is usually associated with characteristic epigenetic signatures at histone tails prevailing as enhancers (e.g. H3K27ac), promoters and gene bodies as indicated. (**B**) Schematic representation of proximity ligation assays (PLA) combined with single molecule (sm)RNA-FISH to monitor the NF-κB status at the single cell resolution. High numbers of NF-κB:IκBα dimers (red dots) and low numbers of smRNA-FISH signals (green dots) characterize cells with a silenced NF-κB pathway, while low numbers of NF-κB:IκBα dimers and high numbers of smRNA-FISH signals characterize cells with high NF-κB activity. For details see text. NF-κB (left sequence logos) and AP-1 (right sequence logos) motifs were obtained from the JASPAR data base (https://jaspar.genereg.net).

In the long-term, the NGS-based approaches should be complemented by advanced proteomic methods that allow to determine and quantify secreted proteins and proteins in body fluids, such as proximity extension assays (PEA) or adapted liquid chromatography mass spectrometry set ups (LC-MS/MS) (Geyer et al., 2019; Petrera et al., 2021). With respect to the importance of the activation status of the NF-κB system for the TME, proximity ligation assays (PLA) can be combined with immunofluorescence and single-molecule RNA fluorescence in situ hybridization (smRNA-FISH) to monitor the flow of signal transduction, that is the formation of active NF-κB dimers and the nuclear translocation as well as the resulting expression of NF-κB target genes in single cells (Figure 5; Mayr-Buro et al., 2019; Meier-Soelch et al., 2021).

In conclusion, the combined application of NGS and proteomics methods will allow to (i) deconvolute the main cellular sources of the altered cytokine milieu and (ii) quantify the entire spectrum of mediators that drive TME phenotypes as a prerequisite for much more precise targeted therapies.

## Clinical relevance

### Biomarkers

Most lung cancer patients are diagnosed at the advanced stage with metastasized tumor. Therefore, application of predictive biomarkers to identify and categorize the lung tumor as early as possible cannot only improve the diagnosis but also increase the efficiency of targeted therapies. Recent advancements made in genomic analyses and onco-immunology revealed two classes of predictive biomarkers in NSCLC; first, the presence of druggable driver oncogenes such as *EGFR* mutations and anaplastic lymphoma kinase (*ALK*) rearrangements and second, TME-based biomarkers including immune checkpoint molecules (Villalobos and Wistuba, 2017; Kerr et al., 2021). However, although the usage of these biomarkers has improved diagnosis and patient survival, the majority of NSCLC patients does not respond or develops resistance to targeted therapies.

Given the crosstalk between the cancer cells and TME, both the cancer cell genetic landscape and immune cell profile determine the efficacy of targeted therapies. Thus, the combination of tumor genetic landscape and immune checkpoint profile together can be helpful to define the best candidates for immune therapy. The best example for the success of this approach is the FDA approval of pembrolizumab (anti PD-1), for the treatment of adult and pediatric patients with unresectable or metastatic tumor with TMB-H ≥10 (mut/Mb) and solid tumors (Marcus et al., 2021). A recent meta-analysis of patients who received PD-L1/PD-1 therapies has shown high TMB (≥10 mut/Mb) to be significantly correlated with prolonged progression free survival (PFS) compared to patients with low TMB (Zhu et al., 2019). Therefore, the combination of TMB with other TME markers such as PD-L1 level, CD8^+^ tumor infiltrating lymphocytes and MHC profile can help select the best possible candidates that will benefit from PD-L1/PD-1 therapy. Despite these evidences, there are still significant hurdles to overcome including technical challenges in measuring TMB, a general lack of agreement for TMB cutoff and the absence of a standardized method (Addeo et al., 2021) which need to be addressed and resolved prior to future application in clinical routine.

In addition, driver mutation status can also be considered a promising biomarker, especially for exclusion of inefficient treatment strategies. For example, NSCLC patients with *EGFR* mutation showed an unfavorable response to PD-L1/PD-1 inhibitors compared to those with wild-type *EGFR,* which could be related to the low TMB of *EGFR*-mutant tumor and an immunosuppressive TME (Dong et al., 2017b).

More recently, HLA-I LOH has been shown to be a negative predictor of overall survival in non-squamous NSCLC patients treated with ICIs. Interestingly, combining TMB and HLA-I LOH improved the prediction of survival, which suggests a better subcategorization of patients that will benefit from immunotherapies (Montesion et al., 2021). Recent advances in imaging techniques such as multiplex immunofluorescence staining was instrumental in demonstrating the high potential of TME immune phenotype as a prognostic factor. Our group have shown that lower density of anti-tumor M1-like macrophages and higher proximity of cancer cells to pro-tumor M2-like macrophages are associated with poor survival in NSCLC (Zheng et al., 2020b). We also demonstrated that accumulation of Th9 and Th17 cells in lung tumors are correlated with poor survival in lung cancer patients (Salazar et al., 2020). The combination of multiplex immunofluorescence staining and genomic analysis thus may prove a robust predictive tool for the subcategorization of patients.

### Combination therapy

Although the development of lung cancer therapy from cytotoxic chemotherapies to genetic- and immune checkpoint-based strategies has shifted lung cancer therapy toward precision medicine, the study of the crosstalk between genomic landscape and TME immune phenotype offers new possibilities for more advanced personalized treatments. For example, *LKB1* mutation plays a key role in primary resistance to the PD-1 axis blockade in *KRAS*-mutant lung adenocarcinoma (Skoulidis et al., 2018). Interestingly, it has been shown that loss of *LKB1* increases the sensitivity to energetic stress triggered by metformin and phenformin (Shackelford et al., 2013). Moreover**,** the enhanced dependence on the CPS1-associated pyrimidine pool in *KRAS/LKB1* mutated cells suggested a higher sensitivity of this cell type to DNA replication stress, which can lead to DNA damage and cell death (Kim et al., 2017). Therefore, targeting the metabolic vulnerabilities of *LKB1* mutated cancer cells may reverse the resistance of PD-1 blockade therapy in lung adenocarcinoma patients with *KRAS/LKB1* mutation. Regarding *EGFR* mutation, The ADAURA study, a randomized, double-blind clinical phase 3 trial, showed that adjuvant therapy with osimertinib, a third-generation EGFR-tyrosine kinase inhibitors (TKI), significantly improved the disease-free survival among patients with stage IB to IIIA EGFR mutation–positive NSCLC (Wu et al., 2020). Although, osimertinib can induce PD-L1 protein degradation and reduce PD-L1 mRNA expression in vitro, to date, the existing clinical data regarding PD-L1 association with osimertinib in EGFR-mutant NSCLC patients is contradictory. For example, Brown et al. demonstrated that efficacy of osimertinib in the first-line treatment of EGFR-mutated metastatic NSCLC was unaffected by PD-L1 expression (Brown et al., 2020). In contrast, Hsu et al. reported the strong PD-L1 expression in advanced EGFR-mutant NSCLC tumors to be associated with a significantly poorer prognosis in patients that received osimertinib as their first-line EGFR-TKI treatment (Hsu et al., 2022). This highlights the need for further studies to increase our knowledge about the effect of osimertinib on EGFR-mutated TME before and after therapy, which will ultimately improve the subcategorization of EGFR-mutant NSCLC patients.

Further, EGFR-TKIs can also induce a rapid and temporary increase of cytotoxic CD8^+^ T cells, dendritic cells, and a reduction of the pro-tumor M2-macrophage population in EGFR-driven lung tumor mouse models. However, most of these anti-tumor effects gradually diminished with the continuation of treatment while the main immunosuppressive cell type, MDSCs, was consistently becoming more dominant during tumor development under treatment (Jia et al., 2019). Combining EGFR-TKIs with depletion of MDSCs, for example by gemcitabine (Le et al., 2009), may therefore improve the efficiency of treatment.

Another strategy, which has promising effects in lung tumor reduction, is the reprogramming of pro-tumor to anti-tumor macrophages (Zheng et al., 2017). Chemotherapy-resistant *KRAS* patients have a large population of pro-tumor M2-macrophages that support the formation of the immunosuppressive TME phenotype (Katopodi et al., 2021), whereas the re-programming of M2-protumor macrophages through modulation of the Wnt/β-catenin pathway reduced primary and metastatic lung tumors (Sarode et al., 2020). Therefore, the modulation of immune components of the TME and its effect on the mutational landscape of cancer cells may not only improve oncoprotein-targeted therapies but also TME-based therapies such as ICIs.

## Conclusion and future perspective

Over the last decades, the conceptual picture of a tumor has shifted from a solid mass of tumor cells to a complex and dynamic micro-organ where genetically altered cancer cells are embedded into an interactive tumor microenvironment containing numerous non-transformed immune and stromal cell types. This conceptual shift introduces the mutational landscape of the cancer cells and the cellular architecture and phenotype of the TME as the two major determinants of tumor initiation, progression and metastasis. Based on the accumulated and emerging evidence, we propose that the fate of individual tumor subclones depends on how well the cancer cell’s genetic profile and the TME phenotype harmonize. While cancer cells require genomic instability to bring forth strong oncogenic drivers that enable aggressive proliferation, the neoantigens, generated in this process, render the cells vulnerable to immune attack. A balance between these opposing forces is required for optimal tumor growth and results from a mutual crosstalk in which the genetic alterations of the cancer cells induce immunosuppressive signals that shape a tumor-supportive TME, while the TME immune components edit the genetic profile of the tumor cells by depleting highly immunogenic subclones until a balance between the two processes is achieved.

In light of the key roles of cancer cell mutations and the TME phenotype during tumor evolution, integrating these two aspects for lung tumor stratification is expected to help anticipate primary and acquired therapy resistance, the key remaining obstacles to a long-term survival benefit under treatment with targeted and immunotherapy regimens. The main and immediate challenge for the integration of genetic landscape and TME phenotype is the heterogeneity of tumor tissues, which calls for spatially resolved single-cell analysis techniques. To address the TME phenotype and its heterogeneity, highly multiplexed imaging technologies and computational tools have been developed that can quantitatively and at single-cell resolution reveal the spatial distribution of tumor, immune and stromal cell components, their interactions and activity states in distinct tumor niches. While the mutational landscape is still primarily profiled by next generation sequencing of bulk tumor tissue, recent advances with single-cell genomics raise hope that tumor mutational status and TME phenotype can soon be better integrated in one framework for a refined classification of lung tumors that better informs clinical decision making for the benefit of long-term survival.
